# CV1 Chimpanzee Adenovirus Efficiently Transduces Mesenchymal Stem and Leukemia Cells: Implications for Cellular Targeting and Vector Tropism

**DOI:** 10.3390/cancers18020220

**Published:** 2026-01-09

**Authors:** Lorella Tripodi, Maria Vitale, Barbara Izzo, Filippo Scialò, Barbara Lombardo, Lucio Pastore

**Affiliations:** 1 Dipartimento di Medicina Molecolare e Biotecnologie Mediche, Università degli Studi di Napoli Federico II Via Sergio Pansini 5, 80131 Napoli, Italy; lorella.tripodi@unina.it (L.T.);; 2 CEINGE-Biotecnologie Avanzate Franco Salvatore, Via Gaetano Salvatore 486, 80131 Napoli, Italy; vitalema@ceinge.unina.it

**Keywords:** chimpanzee adenovirus, neutralizing antibody, leukemic cells, human mesenchymal stem cells, biodistribution

## Abstract

**Objectives:** Adenoviruses (Ads) are among the most used vectors for gene therapy; human Ad serotype 5-derived (HuAd5) vectors are the most frequently used for gene transfer applications. However, Ad5 infection is endemic in humans, and 20% of the Western population has neutralizing antibodies (nAbs). Pre-existing immunity against HuAd5 represents a major issue for many gene therapy applications. In our study, we evaluated several Ad serotypes derived from chimpanzees (ChAds) in vitro and in vivo to assess their transduction efficiency in various cell types and tissues. We aimed at identifying Ad serotypes able either to transduce “challenging” cell types or to represent a possible alternative to Ad5-derived vectors with comparable infectivity and tropism. **Methods:** We evaluated the efficacy of transduction of twelve ChAds vectors expressing enhanced green fluorescent protein (EGFP) in human embryonic kidney cells, as well as human leukemic and human mesenchymal stem cells, using flow cytometry to determine the percentage of EGFP-expressing cells and their mean fluorescent intensity (MFI). We observed the highest transduction efficiency in the serotype CV1 ChAd; therefore, we proceeded to evaluate toxicity and biodistribution in vivo. **Results:** After in vitro evaluation of twelve ChAds serotypes, we observed that the CV1 serotype was the most efficient in transducing both leukemia cell lines (HL-60 and NB-4) and human mesenchymal stem cells. Furthermore, in vivo analysis of the CV1 serotype induced an inflammatory reaction similar to what was observed after HuAd5 administration. **Conclusions:** ChAds vectors represent an effective alternative for the transduction of cells resistant to HuAd5 infection, such as mesenchymal stem cells and leukemic cells. In addition, we observed that the CV1 ChAd serotype presented a transduction profile similar to HuAd5 in vitro and induced a similar inflammatory response in vivo; therefore, CV1 ChAd-derived vectors represent an interesting alternative for gene therapy applications.

## Simple Summary

Human Ad5 (HuAd5) is the most commonly used vector for gene therapy. However, pre-existing immunity against HuAd5 in humans can limit its effectiveness. As an alternative vector, in our study, we evaluated twelve chimpanzee-derived adenovirus (ChAd) serotypes for their ability to transduce challenging cell types. In vitro, we assessed transduction efficiency using enhanced green fluorescent protein in human embryonic kidney cells, two leukemic cell lines, and human mesenchymal stem cells. Among the serotypes tested, CV1 demonstrated the highest transduction efficiency across leukemia and mesenchymal stem cells. In vivo, CV1 induced an inflammatory response comparable to HuAd5, with no evident increase in toxicity. These findings suggest that CV1 is a promising candidate for gene therapy, offering efficient transduction of cells resistant to HuAd5 and a safety profile suitable for further development.

## 1. Introduction

Adenoviruses (Ads) are pathogen viruses in several species; they usually cause mild infection of the respiratory or gastric tract and more than 51 human serotypes have been described to date. Ads are non-enveloped, icosahedral, double-stranded DNA viruses discovered for the first time in 1953 [1,2]. The main vectors for gene delivery are derived from the human Ad serotype 5 (HuAd5). First-generation (FG)-Ad vectors, lacking the E1 gene or less frequently the E3 gene, can be engineered, produced easily at high titers, and have been proven to efficiently transduce a wide number of cell types. Furthermore, it has also been used for the development of cancer vaccines due to their ability to induce a potent antigen-specific immune response [3,4,5,6]. HuAd5 has been used to develop helper-dependent (HD)-Ad vectors [7,8,9,10,11] devoid of all viral ORFs and is able to transduce hepatocytes with an extremely prolonged duration of expression, correcting the phenotype in several animal models of diseases [8,12,13,14]. A main obstacle in using HuAd5-derived vectors is the high proportion of the human population that has been infected by this virus, developing nAbs [15]; indeed, in America, 40% to 60% of the population has developed detectable nAbs against HuAd5 [16]. These specific antibodies against HuAd5 impair the infectivity of HuAd5-derived vectors, reducing their efficacy [15]. Pre-existing immunity has been addressed in several ways, including capsid chemical modification [9,10,17] and the development of vectors based on alternative serotypes to which the human population is less exposed, including those of chimpanzee origin [18]. Among them, ChAds show advantageous features: (i) low seroprevalence in the human population; (ii) low cross-neutralization in sera against HuAds; (iii) easy production in human cell lines, such as human embryonic kidney 293 cells (HEK 293); (iv) immune responses that are similar to HuAd5, even in the presence of NAbs against ChAds [19,20]. Concerning the immune response, the HuAds or ChAds belonging to groups C and E are considered the most potent group in terms of stimulation of the immune system compared to HuAds and ChAds belonging to group B. Although the mechanism of the distinct immunological potency is not yet elucidated, in vivo, it is hypothesized that the interaction with host receptor and/or viral tropism is involved. Therefore, the Ads belonging to groups C and E use the Coxsackievirus and adenovirus receptor (CAR) to enter host cells while the Ads belonging to group B are able to recognize the CD46 receptor. In addition, Ads of group B are able to transduce alternative tissues like hematopoietic stem cells (HSCs) and dendritic cells (DCs) that are refractory to infection by commonly used adenovirus, becoming attractive gene therapy vectors [21,22]. To date, ChAds have been used in clinical trials for various disease treatments, with successful results [23,24,25,26] confirming that ChAds have ideal features as vaccine platforms. The present study was designed to test the hypothesis that non-human adenoviral serotypes may exhibit distinct tropism and safety profiles compared with commonly used human adenoviruses, potentially offering advantages for vector development. We have characterized 12 FG-ChAd vectors and compared them to FG-HuAd5-. We evaluated the ability of these vectors to infect different cell lines, such as HEK 293 and the leukemic cell lines HL-60 and NB-4. In addition, in our study, we also evaluated the efficiency of transduction in human mesenchymal stem cells (hMSCs). HuAd5-derived vectors do not infect these cells efficiently; therefore, identification of a serotype that allows more efficient transduction has relevant biotechnological implications. Finally, we have selected the CV1-derived vector that had the highest infectivity in the above-mentioned cells.

## 2. Materials and Methods

### 2.1. Cell Lines and Reagents

HEK 293 (human embryonic kidney cells), HL-60 (acute myelogenous leukemia), and NB-4 (acute promyelocytic leukemia) were purchased from the American Type Culture Collection (ATCC, Manassas, VA, USA). HEK 293 cells were cultured in Minimum Essential Medium Eagle Alpha modification (Cambrex Bio Science Verviers, B-4800, Verviers, Belgium). HL-60 and NB-4 were cultured in RPMI 1640 medium (Cambrex Bio Science Verviers, B-4800, Verviers, Belgium). hMSCs were isolated via needle medullary aspiration and kindly provided by Dr Ferdinando Frigeri (Istituto Nazionale tumori, Fondazione S. Pascale, Hematological division, Naples, Italy). The bone marrow sample was washed with PBS enriched with 2% FBS. hMSCs were isolated via the Ficoll (Ficoll-Paque^TM^ PLUS, Avantor, Spring House, PA, USA) gradient: briefly, the sample was centrifuged at 1200 rpm for 30 min and the cell band corresponding to hMSCs between the aqueous layer and Ficoll was collected. Cells were washed twice with PBS with 2% FBS and centrifuged at 1000 rpm for 10 min. The cell pellet was suspended in 10 mL of alfa-MEM (Gibco, Thermo Fisher Scientific, Waltham, MA, USA). All media were supplemented with 10% FBS, 1% L-glutamine, 1% of penicillin (100 U/mL), and 1% streptomycin (100 U/mL); cells were maintained at 37 °C in 5% CO_2_ in a humidified incubator [27]. Infections with Ad vectors were performed in all cell lines following the same procedure: 2 × 10^6^ cells were infected with two viral doses of 3000 vp/mL and 5000 vp/mL. All infections were performed in technical duplicate [28] and in technical triplicate for hMSCs infected with a single viral dose of 6000 vp/mL.

### 2.2. Virus Amplification and Purification

ChAds and HuAd5 viruses were amplified by infection of HEK 293 cells, a cell line complementing E1, in four triple flasks at 60–70% of confluence. Cells were collected after the initial cytopathic effect (CPE) at 48 h, centrifuged at 600 rpm per 15 min, put under three freeze–thaw cycles, and successively centrifuged for 4000 rpm per 25 min. The supernatant was collected, treated with DNase 1 U/µL (Thermo Fisher Scientific, Waltham, MA, USA) and then subjected to two rounds of ultracentrifugation in CsCl (Roche, Switzerland) gradient (2.5 mL of 1.45 gr/mL and 3 mL of 1.25 gr/mL CsCl solutions) [8]. The viral band was collected and dyalized against a 10 mM Tris-HCl pH 8.0, 2 mM MgCl_2_ dialyzing solution (TM). After 2 h, TM was replaced with 1 L of freezing solution (10 mM Tris-HCl pH 8.0, 2 mM MgCl_2_, 4% sucrose) overnight. Finally, viruses were collected, aliquoted, and stored at −80 °C. Viral titer was determined as the number of viral particles (vp)/mL measuring absorbance at 260 nm. DNA concentration of purified viruses was estimated by measuring the absorbance at 260 nm (Table 1). Viral infections in HEK 293 cells and in leukemia cell lines HL-60 and NB-4 were performed, testing two groups of ChAds in two separate experiments. The first group included ChAd3, ChAd5, ChAd6, ChAd7, ChAd9, ChAd10, and HuAd5 serotypes and the second one included ChAd16, ChAd24, CV32, CV33, CV68, and CV1 serotypes. For both groups, two viral doses of 3000 vp/cell and 5000 vp/cell were tested. Viral infection in hMSCs was performed by testing one group of ChAds: HuAd5, ChAd24, ChAd5, ChAd7, ChAd9, ChAd10, ChAd16, ChAd3, ChAd6, CV32, CV33, CV68, and CV1 at 6000 vp/cell. Uninfected cells were used as control in all viral infections.

### 2.3. Flow Cytometric Acquisition and Data Analysis

Infected cells (2 × 10^6^ cells) were collected in 15 mL tubes and centrifuged at 1000 rpm for 10 min. Pellets were then washed with 10 mL of PBS and centrifuged at 1000 rpm for 10 min, as described elsewhere [28,29,30]. Finally, supernatants were removed and pellets were washed with 0.5 mL of PBS in collection tubes for flow cytometric analysis. Determination of cells expressing the enhanced green fluorescent protein (EGFP) reporter gene and evaluation of mean of fluorescence intensity (MFI) were performed using the BD FACSCalibur^TM^ (BD Biosciences, San Jose, CA, USA) and Cell-Quest Pro software version 5.1+.

### 2.4. Animal Experiments

All animal experiments were reviewed and approved by the Experimental Animal Committee of CEINGE Biotecnologie Avanzate Franco Salvatore and the Provincial Government of Italy (n°331/2019-PR-protocol D5A89.35). Seven-to-eight-week-old females C57BL/6J were obtained from Charles Rivers laboratories, Italy. After total anesthesia in an isoflurane chamber, 1 × 10^13^ vp/Kg of ChAd CV1- and HuAd5-derived vectors were administered intravenously (n = 3 per group) into the caudal vein in a total volume of 100 µL; control mice were treated with an intravenous injection with the same volume of PBS. We collected the following biological samples from all treated animals: blood for clinical biochemistry analyses, and spleen and liver samples for flow cytometry and histology. Blood samples (200 μL) were withdrawn at 0, 6, 48, and 72 h after vector administration from the retro-orbital plexus, collected in a 1.5 mL tube containing 20 μL of 50 mM EDTA (pH 8.0) and centrifuged at low speed to obtain the serum.

### 2.5. Blood Biochemistry and Evaluation of Acute Toxicity

Blood count (BC) profile, red blood cell (RBC), white blood cell (WBC), platelets (PLTs), alanine aminotransferase (ALT), and aspartate aminotransferase (AST) were determined by dry chemistry technology on a Vitros 250 Analyzer (Ortho Clinical Diagnostics, Johnson & Johnson Co., Rochester, NY, USA). For quality control, we used two commercial serums (Ortho Clinical Diagnostics) to detect the potential inaccuracy of the methods on both low and high analytes’ concentrations of IL-6 and TNF-alpha levels; they were determined using Bio-Plex cytokine multiplex and analyzed using a Bio-Plex Reader, according to the manufacturer’s instructions (Bio-Rad, Hercules, CA, USA) [31,32]. Briefly, beads coated with antibodies against the selected cytokines were mixed with 10 µL of serum and incubated at room temperature for 1 h. After incubation with streptavidin-PE detection reagent for 30 min and subsequent bead resuspension, the plate was read on the Bio-Plex Reader.

### 2.6. Statistical Analysis

Statistical analyses were performed using the GraphPad Prism 10 software (GraphPad Software, Inc., La Jolla, CA, USA). Data were expressed as mean + standard deviation (SD) or as mean + standard error of mean (SEM). Results were compared by a two-way analysis of variance (ANOVA), and a *p*-value < 0.05 was considered statistically significant. Details about the statistical tests for each experiment can be found in the corresponding figure legends.

## 3. Results

### 3.1. Chimpanzee Adenoviruses Have an Extensive Tropism for HEK 293 Cells

**Figure 1:**
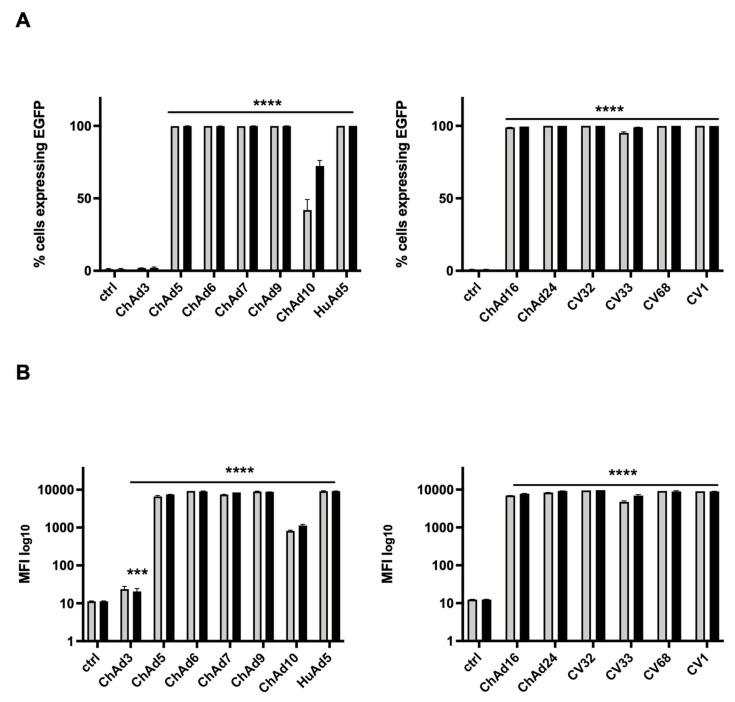
Evaluation of cellular tropism of all chimp adenoviruses and human adenovirus 5 serotypes in HEK 293 cells. Percentage of cells expressing EGFP 72 h after virus infection plotted as mean with SEM (**A**). The statistical significance was evaluated by a two-way ANOVA test followed by Dunnett’s multiple comparison test, applied to suitably transformed percentages by performing an arcsine square root transformation of the percentage values. The mean fluorescent intensity (MFI) of cells expressing EGFP is plotted as mean with SEM, on a log-scale (**B**). The grey bars and black bars in the plots refer to the viral doses 3000 vp/cell and 5000 vp/cell, respectively. The statistical significance was evaluated by a two-way ANOVA test followed by Dunnett’s multiple comparisons test applied on the log-transformed MFI values. The asterisks indicate statistical significance (**** *p* < 0.0001; *** *p* < 0.001) compared to untreated cells (ctrl). The experiments were performed in duplicate for each viral dose (3000 vp/cell and 5000 vp/cell) and the evaluation in HEK 293 was replicated twice.

We amplified and purified 13 Ad vectors expressed EGFP, 12 ChAd-derived FG-Ad vectors kindly supplied by research institute IRBM (Pomezia, Rome, Italy), and one HuAd5-derived FG-Ad. All vectors are proprietary constructs of IRBM. We evaluated the transduction efficiency of all vectors in the HEK 293 cell line. For this purpose, we infected HEK 293 cells with two different viral doses, 3000 vp/cell and 5000 vp/cell. We determined the number of EGFP-expressing cells (Figure 1A) and the relative MFI by cytofluorimetric analysis (Figure 1B). The first group included the following ChAds: ChAd3, ChAd5, ChAd6, ChAd7, ChAd9, ChAd10, and HuAd5 serotypes. The second group included ChAd16, ChAd24, CV32, CV33, CV68, and CV1 serotypes. For both groups, uninfected cells were used as control. The ChAd5, ChAd6, ChAd7, ChAd9, HuAd5, ChAd16, ChAd24, CV32, CV68, and CV1 serotypes infected 100% of cells as confirmed by the high values of MFI ranging from 7000 to 10,000. Results that were statistically significant (*p*-value < 0.0001) and independent from the viral dose were used. Only two serotypes showed a lower level of infection; in fact, ChAd10 infected about 42% of cells and the value of MFI, relative to the lower viral dose used (3000 vp/cell), was about 819, relative to the higher viral dose used (5000 vp/cell). ChAd10 infected about 72% of cells and the value of MFI was about 1100. Moreover, ChAd3 showed infectivity in only 2% of cells with 23.62 and 20.25 MFI values, related to both viral doses used. All differences observed were statistically significant compared to the untreated cells.

### 3.2. CV1 Serotype Showed the Best Tropism in Leukemia Cell Lines

**Figure 2:**
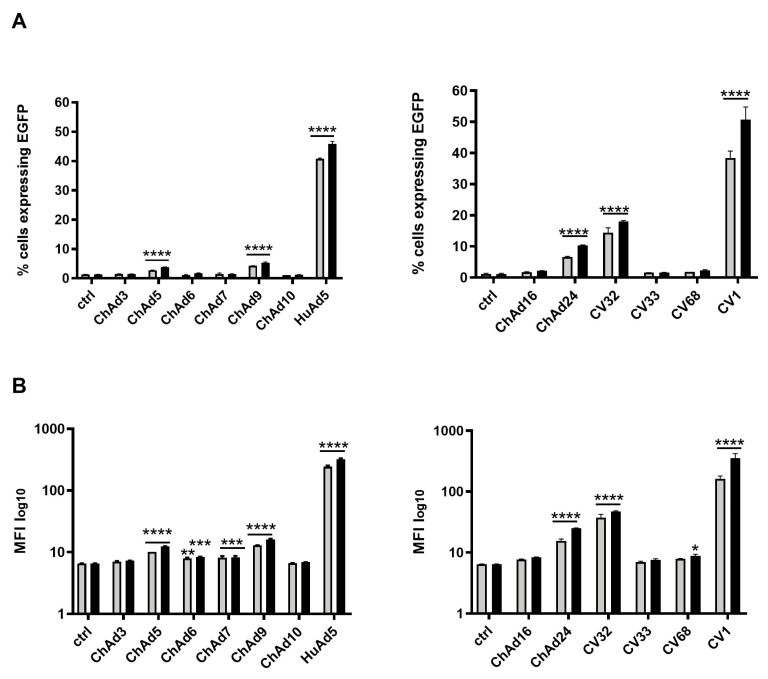
Evaluation of cellular tropism of all chimp adenovirus and human adenovirus 5 serotypes in leukemia cancer cells HL-60. Percentage of cells expressing EGFP 72 h after virus infection plotted as mean with SEM (**A**). The statistical significance was evaluated by a two-way ANOVA test, followed by Dunnett’s multiple comparison test applied to suitably transformed percentages by performing an arcsine square root transformation of the percentage values. The mean fluorescent intensity (MFI) of cells expressing EGFP is plotted as mean with SEM, on a log-scale (**B**). The grey bars and black bars in the plots refer to the viral doses 3000 vp/cell and 5000 vp/cell, respectively. The statistical significance was evaluated by a two-way ANOVA test followed by Dunnett’s multiple comparison test applied on the log-transformed MFI values. The asterisks indicate statistical significance (**** *p* < 0.0001; *** *p* < 0.001; ** *p* < 0.01; * *p* < 0.05) compared to untreated cells (ctrl). The experiments were performed in duplicate for each viral dose (3000 vp/cell and 5000 vp/cell) and the evaluation in HL-60 was replicated twice.

**Figure 3:**
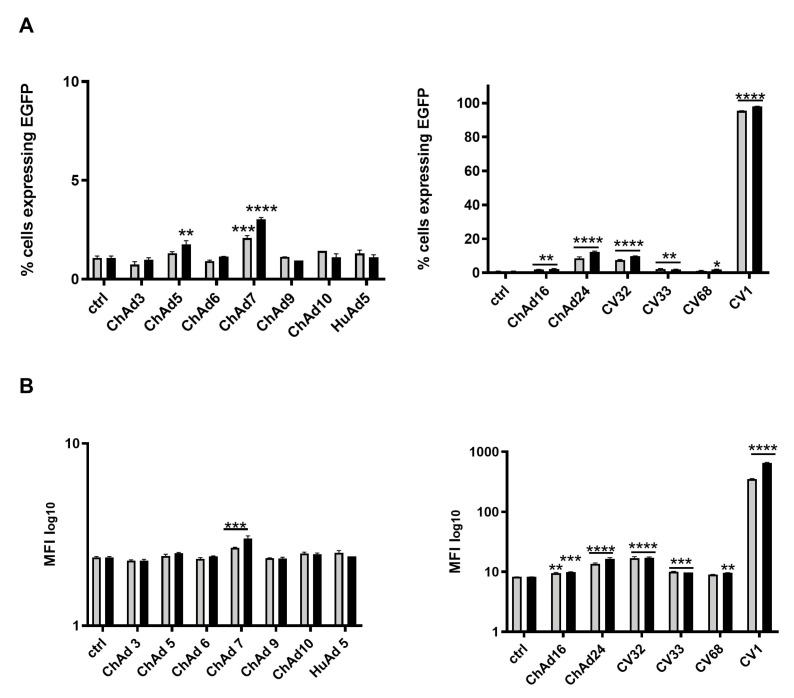
Evaluation of cellular tropism of chimp adenoviruses and human adenovirus serotype 5 in leukemia cancer cells NB-4. Percentage of cells expressing EGFP 72 h after virus infection plotted as mean with SEM (**A**). The statistical significance was evaluated by a two-way ANOVA test, followed by Dunnett’s multiple comparisons test applied to suitably transformed percentages by performing an arcsine square root transformation of the percentage values. The mean fluorescent intensity (MFI) of cells expressing EGFP is plotted as mean with SEM, on a log-scale (**B**). The grey bars and black bars in the plots refer to the viral doses 3000 vp/cell and 5000 vp/cell, respectively. The statistical significance was evaluated by a two-way ANOVA test followed by Dunnett’s multiple comparison test applied on the log-transformed MFI values. The asterisks indicate statistical significance (**** *p* < 0.0001; *** *p* < 0.001; ** *p* < 0.01; * *p* < 0.05) compared to untreated cells (ctrl). The experiments were performed in duplicate for each viral dose (3000 vp/cell and 5000 vp/cell) and the evaluation in NB-4 was replicated twice.

Taking into account the considerable use of gene therapy in the cure of leukemia, we decided to investigate the transducing ability of the ChAds and HuAd5 serotypes, previously tested in the HEK 293 cell line, in two different leukemia cell lines. HL-60 (acute myelogenous leukemia) and NB-4 (acute promyelocytic leukemia) were infected by using two different viral doses (3000 vp/cell and 5000 vp/cell) of ChAds and HuAd5. We determined the number of cells expressing EGFP (Figure 2A) and the relative MFI with a cytofluorimetric analysis (Figure 2B) of all Ads. Given the high number of infections to perform, we divided all ChAds into two groups that were separately investigated, as indicated above. For both groups, uninfected cells were used as control. The CV1 and the HuAd5 serotypes infected about 50% of HL-60 cells at both viral doses used. Compared to CV1, ChAd24 and CV32 showed lower infectivity of about 20–30%, consistent with low values of MFI. Compared to untreated cells, ChAd24 and CV32 showed higher infectivity consistent with high values of MFI, the results of which were both statistically significant. ChAd5 and ChAd9 infected a reduced percentage of cells. All the other ChAds were not able to infect the cells, though the MFI value was statistically significant for ChAd6, ChAd7, and CV68. Similarly, NB-4 cells were infected with all Ads and, as shown in Figure 3, the serotype CV1 infected 100% of cells (Figure 3A), whereas ChAd24 and CV32 serotypes infected 15% of cells, as confirmed by the low values of MFI (Figure 3B). All differences observed results were statistically significant compared to untreated cells.

### 3.3. The Human Mesenchymal Stem Cells Are Efficiently Infected by CV1 and CV32

**Figure 4:**
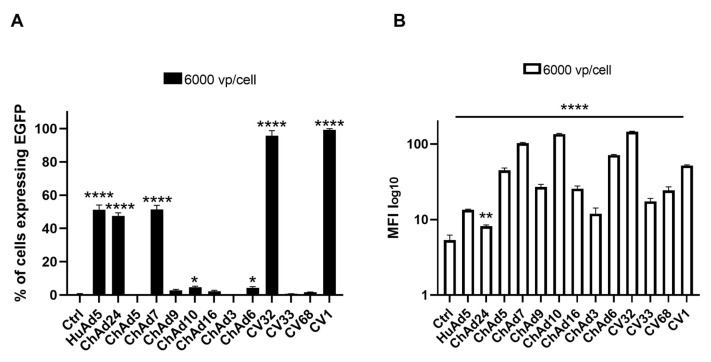
Evaluation of tropism of chimp adenoviruses and human adenovirus serotype 5 in hMSCs. Percentage of cells expressing EGFP 72 h after virus infection plotted as mean with SEM (**A**). The statistical significance was evaluated by a two-way ANOVA test, followed by Dunnett’s multiple comparison test applied to suitably transformed percentages by performing an arcsine square root transformation of the percentage values. The mean fluorescent intensity (MFI) of cells expressing EGFP is plotted as mean with SEM, on a log-scale (**B**). The statistical significance was evaluated by a two-way ANOVA test, followed by Dunnett’s multiple comparison test applied on the log-transformed MFI values. The asterisks indicate statistical significance (**** *p* < 0.0001; ** *p* < 0.01; * *p* < 0.05) compared to untreated cells (ctrl). The experiments were performed in triplicate for the unique viral dose (6000 vp/cell) and the evaluation in hMSCs was replicated twice.

After assessing that certain ChAds efficiently transduced and infected the leukemia cell line, we evaluated the efficacy of transduction of the ChAds in hMSCs, which is a promising therapeutic target for its ability to differentiate in several cell types and their optimization to grow and expand in vitro. However, in order to test the transduction ability of ChAds, we chose the viral concentration of 6000 vp/cell and compared the infectivity with the HuAd5 control virus. The infected hMSCs were harvested 72 h post-infection (hpi) and analyzed by flow cytometer. As performed for previous infections, we evaluated the percentage of the EGFP expressed by infected cells and MFI. As shown in Figure 4A, analysis of EGFP expression demonstrates that HuAd5 infects hMSCs significantly more efficiently than untreated cells, as well as cells transduced with ChAd5, ChAd9, ChAd10, ChAd16, ChAd3, ChAd6, CV33, and CV68 (*p* < 0.0001). In contrast, ChAd24, ChAd7, CV32, and CV1 display transduction efficiencies comparable to HuAd5, indicating a similar ability to infect hMSCs.

Consistent with these findings, Figure 4B shows that although the percentage of EGFP-positive cells is markedly reduced following transduction with ChAd24, ChAd5, ChAd9, ChAd10, ChAd16, ChAd3, ChAd6, CV33, and CV68, the corresponding MFI values do not decrease proportionally. Notably, high MFI values in the context of a low percentage of EGFP-positive cells indicate the presence of a restricted subpopulation of hMSCs with robust EGFP expression. This pattern suggests that, while overall transduction efficiency is limited for these vectors, the cells that are successfully transduced exhibit strong transgene expression, highlighting heterogeneity in viral susceptibility and transgene expression levels within the hMSC population (Flow Cytometry Handbook, 2021). For the percentage of cells expressing EGFP (Figure 4A), we consider them the best delivery vectors analyzed in this study. Though the screening of ChAds revealed that CV1 and CV32 seem to be the best vectors for hMSC transduction (Figure 4), we decided to evaluate toxicity and immuno-response in vivo after assessing the ability of CV1 to transduce and infect efficiently and better than CV32, including the cell lines HEK 293, HL-60, and NB-4.

### 3.4. Preliminary Evidence of Reduced Hepatotoxicity and Mild Immune Response Induced by CV1 in a Mouse Model

**Figure 5:**
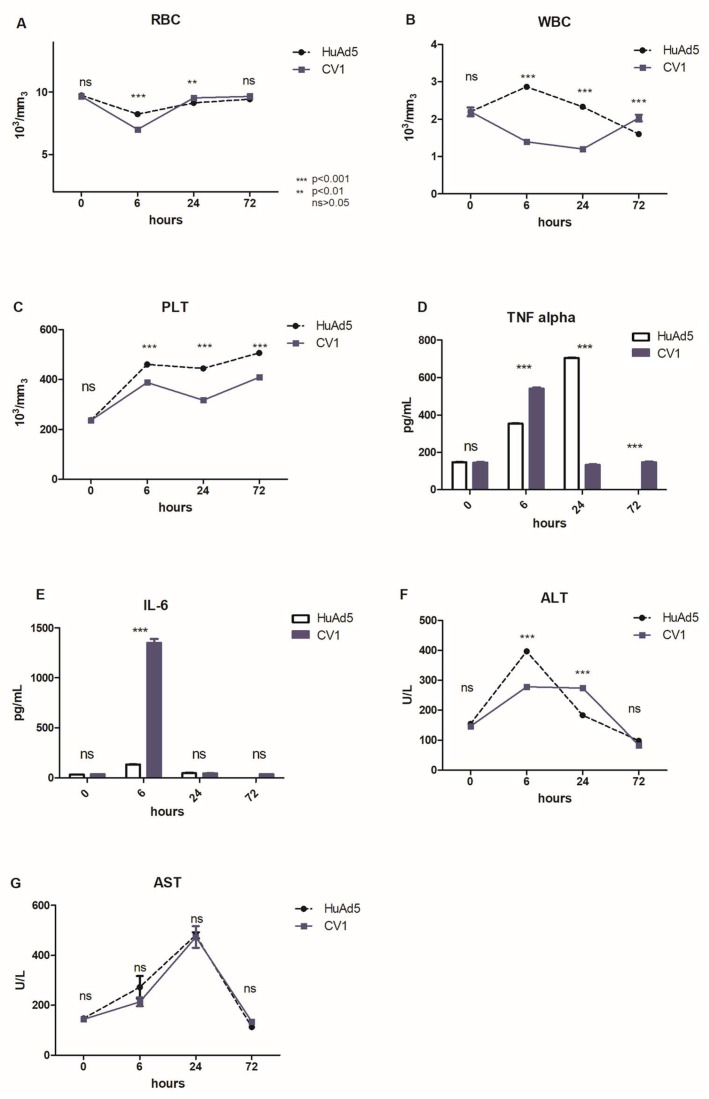
In vivo acute toxicity evaluation and blood count. (**A**) Dosage of red blood cells (RBCs). Dosage of white blood cells (WBCs) (**B**). Dosage of platelets (PLTs) (**C**). Evaluation of TNF-alfa level (**D**). Evaluation of IL-6 level (**E**). Evaluation of Alanine-ammino-transferase (ALT) (**F**). Evaluation of Aspartate ammino-transferase (AST) (**G**). The count was performed in mice treated (n = 3 per group) with CV1 or HuAd5 as the control group and the data reported in the graphic represented the mean ± SEM. The statistical analysis was performed with GraphPad Prism 5.02 version by a two-way ANOVA test. The asterisks indicate statistical significance (*** *p* < 0.001; ** *p* < 0.01) compared to the control group (HuAd5) and ns means not significant means > 0.05. All data were analyzed with a two-way ANOVA test. The acute toxicity evaluation and blood count were repeated twice.

Following the screening of ChAd tropism, we decided to further evaluate the toxicity and immuno-response in a preliminary in vivo evaluation involving three mice for each group. After assessing the ability of CV1 to transduce and efficiently infect in vitro human leukemic and hMCSs, we decided to evaluate its efficacy in vivo. For this purpose, we intravenously administered 1 × 10^13^ vp/Kg of CV1 in seven-to-eight-week-old C57BL/6J female mice and HuAd5 as control. Analysis of the inflammatory response was performed by evaluating the levels of biomarkers of damage as TNF-alfa, IL-6, Alanine aminotransferase (ALT), and Alanine aminotransferase (ALT). General indications on the healthy status of mice after CV1 administration were obtained by analyzing blood samples harvested at 0, 6, 24, and 72 hpi. We performed a count of red blood cell (RBC), white blood cell (WBC), and platelets (PLTs) and, as shown in Figure 5A, there were no differences in RBC count between CV1 and HuAd5 at time 0 (Figure 5A). The main difference is reported at 6 hpi; in mice treated with CV1, a significant decrease in RBC that returned to physiological condition at 72 hpi was observed. The WBC of treated mice decreased significantly at 6 hpi and 24 hpi, while at 72 hpi, it rose compared to the control group (Figure 5B). Furthermore, the PTL count suggests that the treatment with CV1 induces a significant thrombocytopenia, compared to HuAd5 (Figure 5C). Thrombocytopenia is a phenomenon already described in the literature and is symptomatic of Ads infection [33,34,35]. The dosage of TNF-alfa, in the control group, revealed a gradual increase from 6 to 72 hpi, that was extinguished after 72 hpi. Similarly, the administration of the CV1 induced a strong increase after 6 hpi and a gradual shutdown of TNF-alfa response from 24 to 72 hpi (Figure 5D). Furthermore, in Figure 5E, the analysis of IL-6 levels showed a strong response at 6 hpi in CV1-treated mice (Figure 5E). The evaluation of ALT and AST, as the most relevant hepatic markers [36], displayed an increase in ALT level at 6 hpi for both viruses, returning to the normal range of 72 hpi (Figure 5F). The evaluation of AST levels suggests that both Ads showed a similar trend, with growth over time and return to physiological condition after 72 hpi (Figure 5G).

## 4. Discussion

To date, several clinical trials based on the HuAd5 vector are still ongoing since its biology is well-known. However, pre-clinical and clinical trial reports have revealed several drawbacks of this serotype, one of which is the widespread pre-existing anti-HuAd5 immunity in humans due to prior infection in youth [37]. In addition, studies indicated that the coagulation factor X (FX) specifically binds the hexon, the major Ad5 capsid protein, resulting in the direction of HuAd5 to FX receptors abundantly expressed in liver cells. This, in turn, causes massive transduction to the liver [38,39,40]. In order to circumvent the presence of nAbs and the massive transduction to the liver, several solutions can be considered; one involves the use of a helper-dependent (HD)-Ad vector system that, through the covalent modification of capsids (named PEGylation), is able to stimulate a milder immune response, avoiding hepatic retention. Another solution involves alternative sources like non-human primate (NHP)-derived adenoviruses. Increasing numbers of pre-clinical and clinical trials have applied ChAds for vaccine development against various infectious diseases, such as Ebola, HIV, HCV, malaria, rabies, Crohn’s disease, and respiratory syncytial virus (RSV) [23,41,42,43,44,45,46,47], confirming that ChAds have ideal features as vaccine platforms. Based on the evidence, we investigated the tropism of several replication-defective chimeric vectors expressing EGFP. In our study, the adenoviral vector derived from the CV1 serotype turned out to be the best candidate as NHP-derived Ads for its high tropism in HEK 293 cell lines and HL-60 and NB-4 leukemia cells. CV1, which is closely related to B2 subgroup HuAds, unlike other ChAds serotypes, utilizes CD46 for cell entry as a primary cellular receptor, thus infecting cells in a CAR-independent manner [48]. Specifically, the HEK 293 cell, HL-60, and NB-4 leukemia cells expressed high levels of CD46 as reported by the human protein atlas 8 “https://www.proteinatlas.org/ENSG00000117335-CD46/cell+line#leukemia (accessed on 1 July 2025)”. One of the major limitations of HuAds is that the sensitivity and the infection efficiency of target cells depend on CAR expression levels. To demonstrate this, the elevated virus titer has to be used to achieve high infection efficiency in the hMSCs that expressed the CD46 receptor but scarcely expressed CAR [48].

In addition, different receptors’ cellular expression, as well as capsid architecture and fiber protein conformation, may influence initial virus cell interactions, virion stability, and post-entry processing. Moreover, intracellular trafficking events, including endosomal escape, cytoplasmic transport, and nuclear entry, are critical steps that can differ substantially among adenoviral serotypes and affect overall transgene expression. Variations in capsid disassembly kinetics or interactions with host trafficking machinery may further modulate vector efficiency and cell type specificity. Although these mechanisms were not directly investigated in the present study, they represent plausible contributors to CV1 performance and warrant targeted investigation in future studies.

In our study, CV1 and CV32 serotypes are able to infect 100% of hMSCs by using 6000 vp/cell. Despite the modest results obtained in vitro for several ChAds, a future experiment will be refined in order to perform a single experiment with all ChAds and a positive control (HuAd5) that could strengthen the comparative analysis between the two groups of viruses tested, namely leukemia cell lines and hMSCs, as seen in HEK 293. Consistent with our encouraging in vitro results obtained with the CV1 serotype, we evaluated the toxicity profile and immune response triggered by CV1 infection in vivo. After 6 h and 24 h of infection, we detected a significant decrease in RBC and WBC count, compared to the control group treated with HuAd5. Likewise, the injection of CV1 induced a significant decrease in platelet count, suggesting a transient condition of thrombocytopenia that restored to physiological levels 72 h post-infection. The preliminary evaluation of immunogenicity of CV1 serotype suggests its safety profile as indicated by low values of TNF-alfa and IL-6, even though other deeper analyses are needed. In addition, given the low levels of the hepatic markers ALT and AST, CV1 appears to show a trend toward an improved safety profile. However, these observations are preliminary and based on limited experimental evidence. Overall, this exploratory study suggests a potential role for the CV1 serotype in the development of viral vectors for cancer therapy or metabolic diseases. Nonetheless, more extensive and in-depth studies are required to rigorously assess safety, efficacy, and biodistribution before definitive conclusions can be drawn. While CV1 may represent a promising candidate, its suitability as a vector must be confirmed through comprehensive pre-clinical evaluation.

## 5. Conclusions

While CV1 demonstrated notable transduction efficiency in both NB-4 leukemia cells and human mesenchymal stem cells, our results indicate that its tropism is broad rather than strictly tumor-specific. However, these findings highlight CV1 as a promising gene delivery vector with the potential for versatile applications. Further studies will be valuable to explore tumor selectivity, replication competence, and efficacy in relevant in vivo models. Additionally, preliminary in vivo data suggests a favorable hepatotoxicity profile, although these observations are limited and warrant further investigation. Overall, our study supports the potential of CV1 as an efficient and adaptable vector platform, providing a strong foundation for future research aimed at both gene therapy and targeted applications.

## Figures and Tables

**Table 1:** The concentration of DNA of purified viruses was estimated by measuring the absorbance at 260 nm, their relative taxonomical classification of all Chimpanzee adenovirus and human adenovirus 5 serotypes, and the main receptors used to interact with host cells.

Adenoviral Serotypes	Concentrations (vp/mL)	Taxonomical Groups	Receptors
HuAd5	3.3 × 10^11^ vp/mL	C	CAR
ChAd3	5.5 × 10^11^ vp/mL	C	CAR
ChAd5	1.3 × 10^11^ vp/mL	D	CD46
ChAd6	2.8 × 10^11^ vp/mL	E	CAR
ChAd7	2.4 × 10^11^ vp/mL	D	CD46
ChAd9	4.4 × 10^11^ vp/mL	E	CAR
ChAd10	2.0 × 10^11^ vp/mL	E	CAR
ChAd16	4.0 × 10^11^ vp/mL	D	CD46
ChAd24	4.4 × 10^11^ vp/mL	C	CAR
CV32	3.7 × 10^11^ vp/mL	D	CD46
CV33	1.1 × 10^12^ vp/mL	E	CAR
CV68	2.6 × 10^11^ vp/mL	E	CAR
CV1	1.56 × 10^12^ vp/mL	B	CD46; CD80/CD86 Desmoglein-2; Heparan Sulfate Proteoglycans (HSPGs)

## Data Availability

Data are contained within the article.

## Abbreviations

Ads	Adenoviruses
HuAd5	human Adenovirus serotype 5
ChAds	Chimpanzee adenoviruses
hpi	hours post infection
nAbs	Neutralizing antibodies
CAR	Coxsackievirus and adenovirus receptor
hMSCs	human mesenchymal stem cells
DCs	Dendritic cells
EGFP	enhanced green fluorescent protein
IU	Infectivity units
MFI	Mean fluorescent intensity
